# Germline-restricted chromosome (GRC) in the sand martin and the pale martin (Hirundinidae, Aves): synapsis, recombination and copy number variation

**DOI:** 10.1038/s41598-020-58032-4

**Published:** 2020-01-23

**Authors:** Lyubov P. Malinovskaya, Kira S. Zadesenets, Tatyana V. Karamysheva, Ekaterina A. Akberdina, Elena A. Kizilova, Margarita V. Romanenko, Elena P. Shnaider, Mariya M. Scherbakova, Igor G. Korobitsyn, Nikolai B. Rubtsov, Pavel M. Borodin, Anna A. Torgasheva

**Affiliations:** 1 Institute of Cytology and Genetics, Russian Academy of Sciences, Siberian Department, 630090 Novosibirsk, Russia; 2 Novosibirsk State University, Novosibirsk, 630090 Russia; 3 SibEcoCenter LLC, Novosibirsk, 630009 Russia; 4 Tomsk State University, Tomsk, 634050 Russia

**Keywords:** Evolutionary genetics, Cytogenetics

## Abstract

All songbirds studied to date have an additional Germline Restricted Chromosome (GRC), which is not present in somatic cells. GRCs show a wide variation in genetic content and little homology between species. To check how this divergence affected the meiotic behavior of the GRC, we examined synapsis, recombination and copy number variation for GRCs in the closely related sand and pale martins (*Riparia riparia* and *R. diluta*) in comparison with distantly related estrildid finches. Using immunolocalization of meiotic proteins and FISH with GRC-specific DNA probes, we found a striking similarity in the meiotic behavior of GRCs between martins and estrildid finches despite the millions of years of independent evolution. GRCs are usually present in two copies in female and in one copy in male pachytene cells. However, we detected polymorphism in female and mosaicism in male martins for the number of GRCs. In martin and zebra finch females, two GRCs synapse along their whole length, but recombine predominately at their ends. We suggest that the shared features of the meiotic behavior of GRCs have been supported by natural selection in favor of a preferential segregation of GRCs to the eggs.

## Introduction

Most multicellular organisms have the same chromosome set in all their cells. Songbirds are an exception. In their germ cells, they have an additional chromosome called “Germline Restricted Chromosome” (GRC), which is not present in somatic cells^1^. The GRC has been described first in the zebra finch^2,3^ and then in the Bengalese finch^4^. In the male germ line cells of these species, the GRC is present in one copy. At meiotic prophase, it forms the lateral element of the synaptonemal complex (SC) and proceeds as a univalent. After meiotic divisions, it is ejected from the spermatocytes as a heteropicnotic body. The GRC is transmitted to the progeny via females. The oocytes of the finches usually contain two copies of GRC, which form normal bivalents and recombine.

The estrildid finch GRC was considered a genetic curiosity, until Torgasheva *et al*.^5^ recently demonstrated that GRCs are widespread among the songbirds. The GRC is present in the germ cells of all the sixteen songbird species studied and is absent from the germline genomes of all species examined in other avian orders. This indicates that the GRC probably evolved in the common ancestor of the songbirds about 35 MYA. The songbird GRCs contain a variety of repetitive elements and unique sequences with paralogs in the somatic genome^5–7^. Some of the genes located at the zebra finch GRC show signals of positive selection. Many of them are expressed in the germ cells^6,7^. These data suggests that acquired genes make GRC a functional element of songbird germline genomes.

The GRCs of different species show a wide variation in genetic content and have little homology between each other^5^. This indicates that the GRC has undergone significant changes in the extant descendant lineages. It remains unclear how these changes have affected the diagnostic features of the GRC, i.e. its sex-specific meiotic behavior and transmission to the progeny. The only two species studied in detail are the closely related zebra finch and Bengalese finch^3,4^. These two species diverged between 9 and 12 MYA^8^. Their GRCs are similar in size and show rather a high level of homology, as was estimated by crosspieces FISH with GRC-derived DNA probes^5^.

In this paper, we examine the meiotic behavior and copy number variation of GRCs in the germ cells of pale martin females and males and sand martin females sampled from natural populations. To visualize the GRCs, we used FISH with GRC-derived DNA probes and immunostaining with antibodies to SYCP3, the main protein of the lateral elements of the SC and MLH1, the mismatch repair protein marking mature recombination nodules.

Sand martins and pale martins contain large acrocentric GRCs^5^. These species diverged about 2 MYA from each other^9^ and about 30 MYA from the zebra finch and the Bengalese finch^8^. Reciprocal crosspieces FISH with species-specific GRC-derived DNA probes between the pale martin and the zebra finch revealed a low homology between their GRCs. Yet the GRCs of martins and estrildid finches are strikingly similar in appearance^5^.

A comparison of the meiotic behavior of the GRC in phylogenetically close vs distant species may shed a light on its evolution. Another advantage of martins as a new model to study GRC is that they are numerous, widespread and live in large breeding colonies. Analysis of the birds from natural populations allows us to estimate GRC variation in copy number and morphology between the germ cells of the same individual, between sibs and unrelated individuals, between sexes of the same species and between closely and distantly related species. The results of this analysis are important for understanding the functional role of the GRC and the patterns of its mitotic and meiotic transmission and elimination.

## Results

### Identification of a GRC

**Figure 1:**
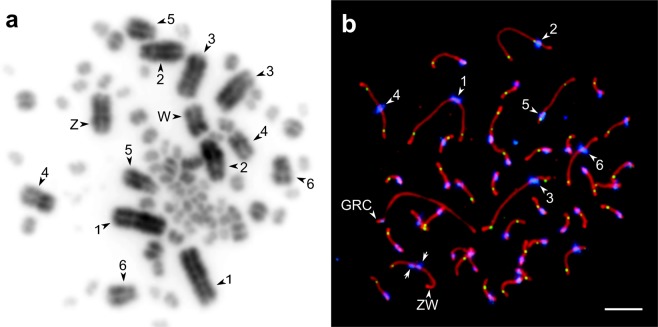
Bone marrow metaphase chromosome (**a**) and SC (**b**) spreads from a sand martin female. Bone marrow chromosomes stained with Giemsa, SC spreads immunolabelled with antibodies against SYCP3 (red), centromere proteins (blue) and MLH1 (green). Arrowheads indicate the largest chromosomes, identified by their size ranks and morphology. Arrows point at the misaligned centromeres of ZW bivalent. Bar – 5 µm.

The somatic karyotypes of the sand martin and the pale martin included 39 pairs of autosomes and a pair of sex chromosomes (Fig. 1a), matching the description of the sand martin karyotype given by Li and Bian^10^. All macrochromosomes in spreads obtained from bone marrow cells were metacentric or submetacentric (biarmed). On all SC spreads of all examined individuals of both species, we observed at least one additional large acrocentric (uniarmed) macrochromosome, which was not present on the bone marrow spreads (Fig. 1b). We identify this chromosome as being a GRC.

**Figure 2:**
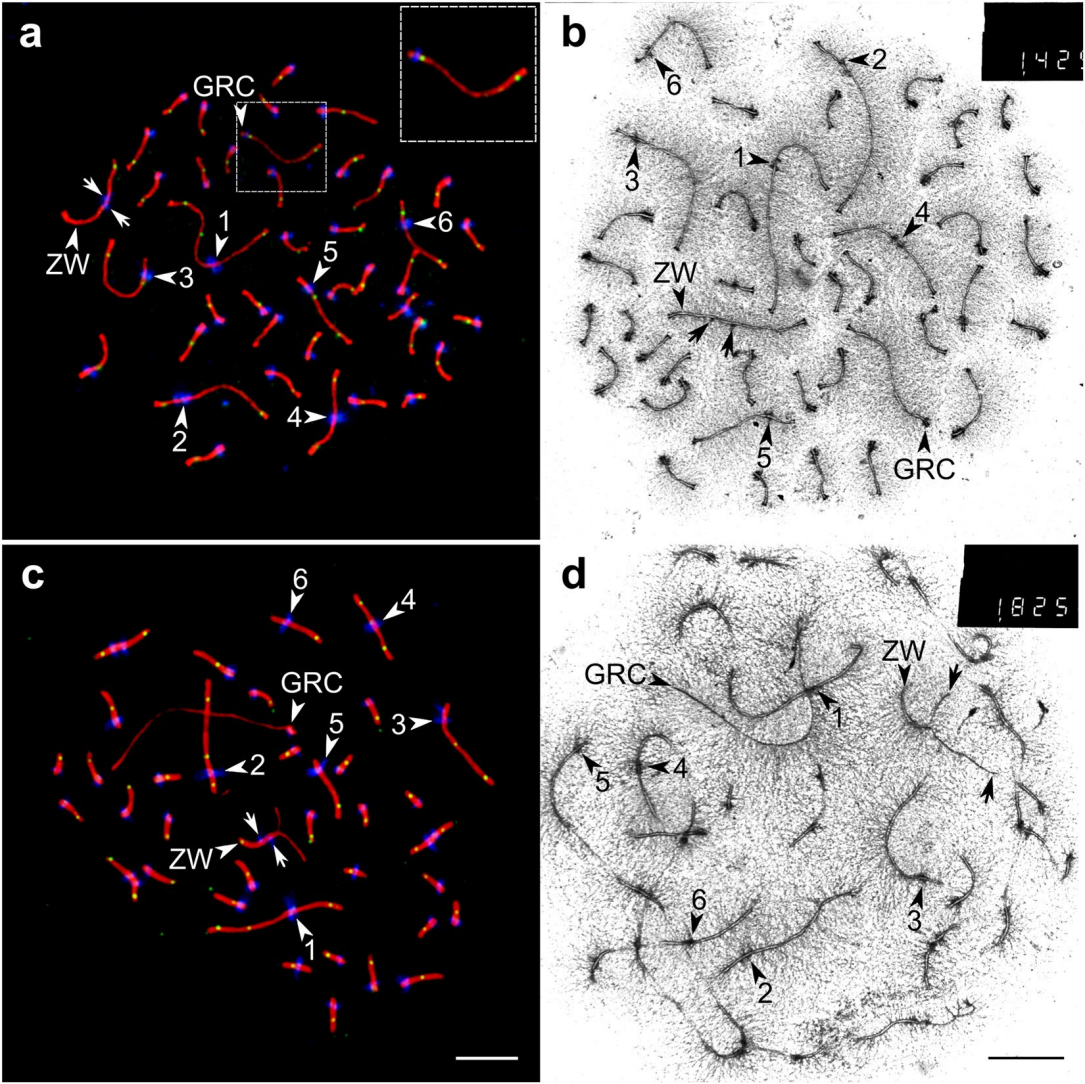
GRC bivalents (**a**,**b**) and univalents (**c**,**d**) in sand martin pachytene oocytes. (**a**,**c**) Fluorescence microscopy images of the pachytene oocytes immunolabelled with antibodies against SYCP3 (red), centromere proteins (blue) and MLH1 (green). (**b**,**d**) Electron microscopy images of the pachytene oocytes after AgNOR staining. Arrowheads indicate the largest chromosomes, identified by their size ranks and morphology. Arrows point at the misaligned centromeres (**a**–**c**) and asynapsed ends (**d**) of ZW axial elements. Bar – 5 µm.

We estimated the number of GRC copies per pachytene cell by the synaptic configurations they formed. Two copies formed a bivalent (Fig. 2a,b), one copy occurred as an univalent (Fig. 2c,d). Bivalents were easily distinguishable from univalents under an electron microscope by the number of the lateral elements (Fig. 2b,d). Under a light microscope, the GRC bivalents appeared just as normal autosomal bivalents (Fig. 2a), while the GRC univalents showed a less intense SYCP3 signal. The lack of the MLH1 signal was another diagnostic feature of the GRC univalents (Fig. 2c).

Analysis of meiosis in zebra finch males^11^ demonstrates that GRCs are usually ejected from the nuclei of secondary spermatocytes and appear as round heteropicnotic bodies nearby. We observed similar round bodies near secondary spermatocytes on the preparations of meiotic chromosomes of pale martins.

### GRC copy number polymorphism in females

In total, we analyzed 1012 oocytes of 24 sand martin and three pale martin female chicks. The GRC was present in two copies in the majority of the females examined (in 20 sand martins and in all pale martins), and in one copy in four sand martin females (Table 1). We detected no mosaicism for GRC copy number among females. All oocytes of each female contained either one, or two GRCs. Female chicks from the same nest tended to have the same GRC number (Table 1). However, more data are needed to verify nest clustering for GRC number.

**Table 1:** Polymorphism for GRC copy number in oocytes of sand martin and pale martin females.

Species	Nest ID	Chicks in the nest	No. females examined	No. individuals with	
				one GRC	two GRCs
Sand martin	1	9	4	0	4
	2	5	3	0	3
	3	3	2	0	2
	4	4	2	0	2
	5	3	2	0	2
	6	4	1	0	1
	7	3	1	0	1
	8	4	1	0	1
	9	3	1	0	1
	10	3	1	1	0
	11	5	2	2	0
	12	6	4	1	3
Pale martin	13	3	2	0	2
	14	3	1	0	1
Total	14	58	27	4	23

GRC bivalents in most pachytene oocytes were completely paired (Fig. 1a,b). We detected only one pale martin pachytene oocyte with asynapsis in the middle of the GRC bivalent. The same oocyte contained the unpaired Z and W chromosomes. Perhaps, the oocyte was at early pachytene, and partial asynapsis of the GRC was temporary. The GRC bivalents did not differ in size between sand martin and pale martin females (12.9 ± 4.3 µm and 11.6 ± 2.7 µm correspondingly; Mann–Whitney *U* test; p = 0.053).

**Figure 3:**
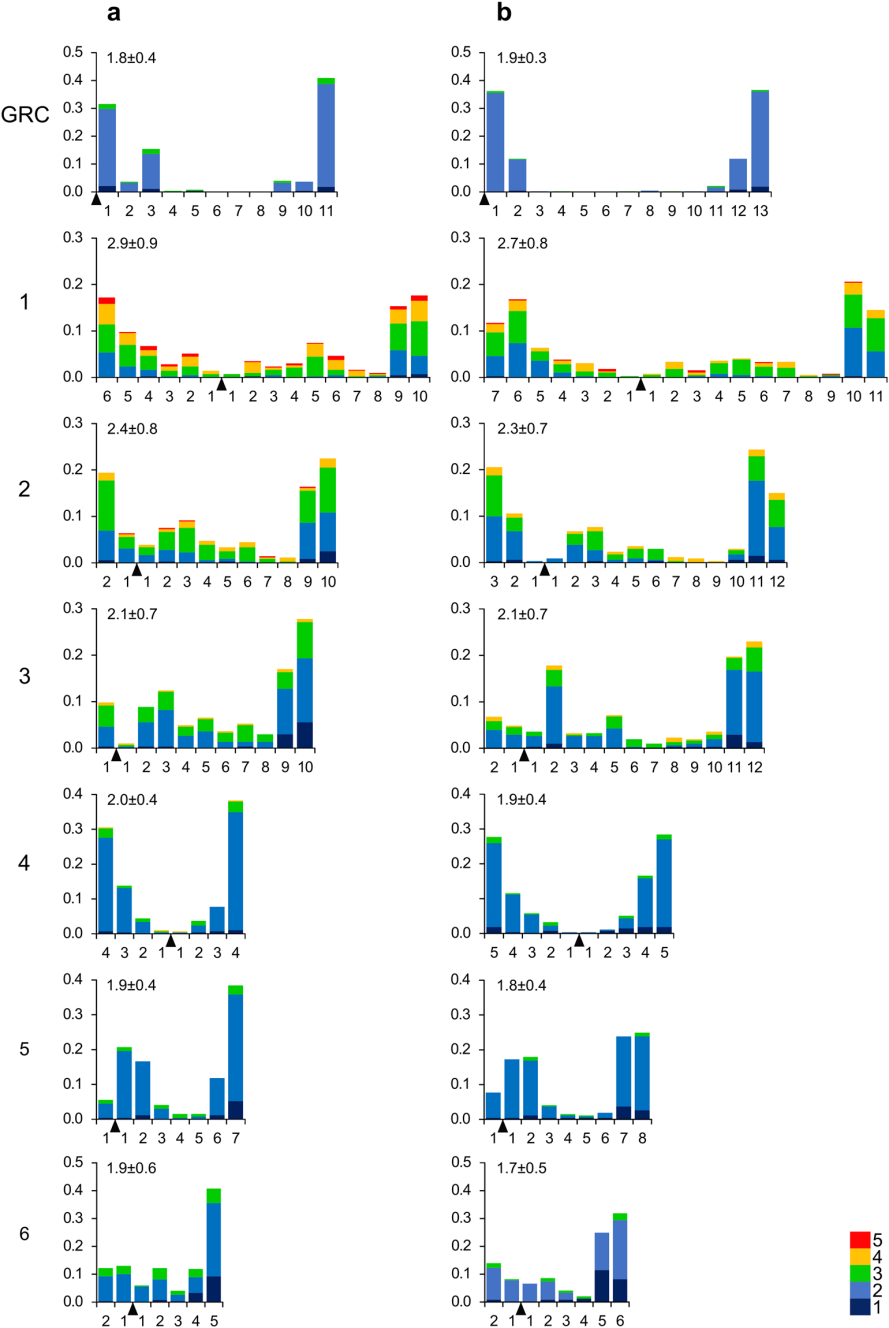
Distribution of MLH1 foci along individual SCs in pachytene oocytes of pale martin females (**a**) and sand martin females (**b**). On the *x*-axis: the relative position of MLH1 foci at the six largest macroSCs and GRC bivalents in relation to the centromere (black triangle). The width of the interval is approximately 1 μm. On the *y*-axis: the proportion of MLH1 focus number in each interval. Colors indicate bivalents with 1–5 MLH1 foci per bivalent. The scale shows the color codes. The numbers to the left of the *y*-axis stand for chromosome numbers; the numbers above each graph show the average number of MLH1 foci at a given chromosome of a given species.

Most GRC bivalents in the martin oocytes contained two MLH1 foci. Bivalents with one and three foci were rare (10.9% and 4.8% in the sand martin and 6.9% and 1.6% in the pale martin). The average number of MLH1 foci per GRC bivalent was the same in sand martin and pale martin oocytes (1.9 ± 0.3 and 1.9 ± 0.4, correspondingly; Mann–Whitney *U* test; p = 0.731). It did not differ significantly from MLH1 foci number at the autosomal bivalents of a comparable size: SC2 and SC3 (p > 0.05). However, the distribution of MLH1 foci along GRC bivalent differed from the distribution along autosomal SCs. The GRC bivalent showed stronger polarization of the MLH1 foci distribution. Most MLH1 foci were located in the distal and proximal deciles of the GRC, while macrochromosomes show more even MHL1 focus distribution (Fig. 3).

GRC univalents did not form foldbacks or hairpin-like configurations (Fig. 2c,d). We did not observe ectopic pairing between GRC univalents and the chromosomes of the basic set. No MLH1 signals were detected at the univalents. The GRC univalents were on average 1.5 times longer than the GRC bivalents (19.3 ± 7.0 µm and 12.9 ± 4.3 µm, correspondingly; Mann–Whitney *U* test; p < 0.001). It is well known that complete synapsis of homologous chromosomes leads to a reversible shortening of the lateral elements of the SC at pachytene in comparison to their unpaired or partially paired state at zygotene and diplotene^12^.

### Polymorphism and mosaicism for GRC copy number in males

In the pale martin males, we detected both polymorphism and mosaicism for GRC number in pachytene cells (Table 2). Two out of nine males had one GRC copy forming a univalent in all pachytene cells examined. Seven males were mosaic for GRC copy number: six for two copies and one for three. In the males mosaic for two copies, a proportion of cells carrying GRC bivalents varied from 2% to 61%. (Table 2). In the male mosaic for three GRC copies, most cells (59 out of 85) contained a GRC univalent, 21 cells had an incompletely paired bivalent and five cells had one univalent and one completely paired bivalent (Fig. 3).

**Table 2:** Mosaicism for GRC number in spermatogonia (SPG) and pachytene spermatocytes (P) of pale martin males.

Individual’s ID	No. cells examined		One GRC		Two GRCs		Three GRCs	
	SPG	P	SPG	P	SPG	P	SPG	P
1	5	77	5	68	0	9	0	0
2	205	62	190	24	15	38	0	0
3	—	66	—	66	—	0	—	0
4	8	58	8	55	0	3	0	0
5	—	55	—	54	—	1	—	0
6	74	85	70	59	4	21	0	5
7	19	111	19	101	0	10	0	0
8	—	26	—	26	—	0	—	0
9	102	52	76	45	26	7	0	0
Total	413	592	368	498	45	63	0	5

**Figure 4:**
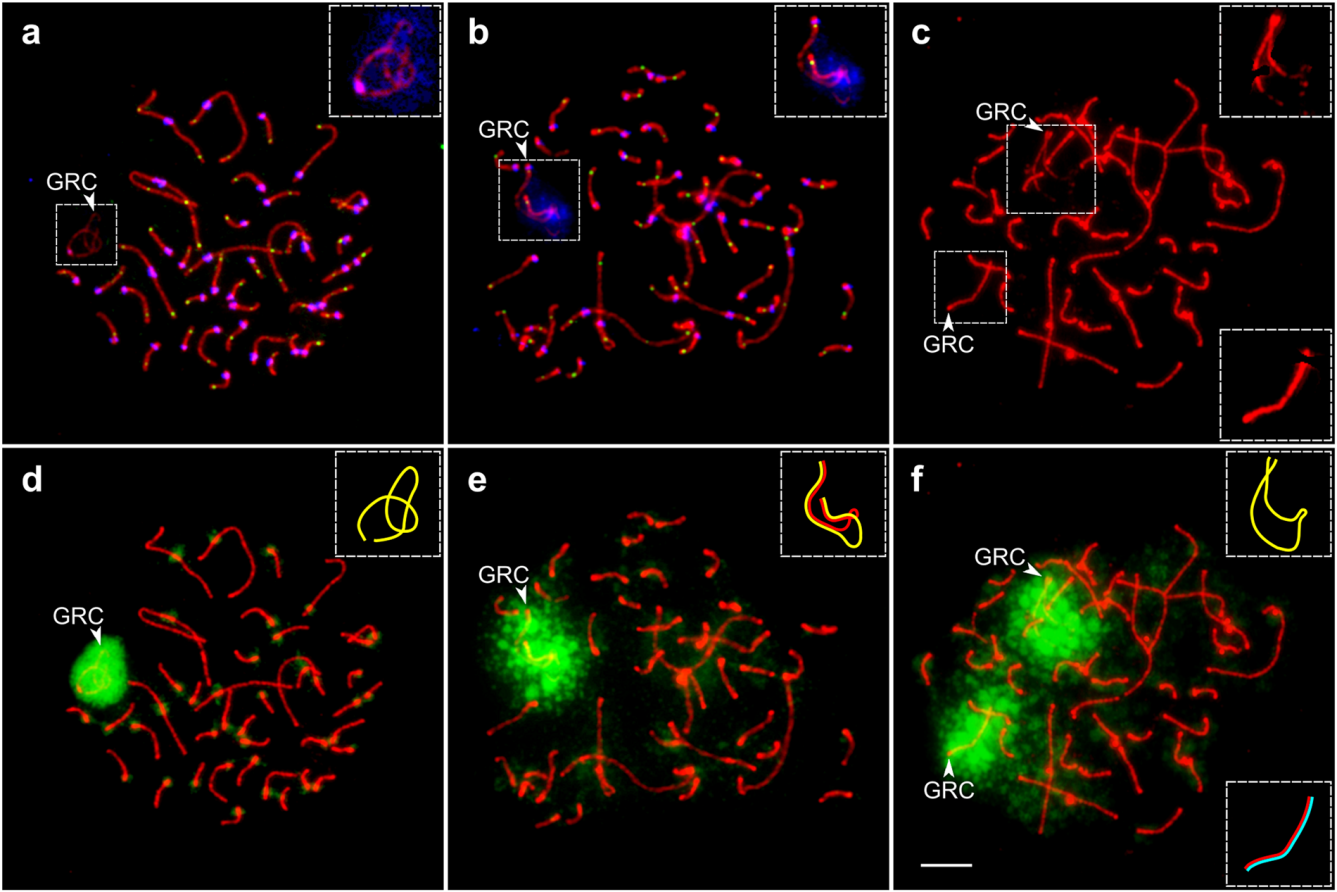
Pale martin pachytene spermatocytes with one (**a**,**d**), two (**b**,**e**), and three (**c**,**f**) copies of GRC. (**a**–**c**) Cells after immunostaining with antibodies against SYCP3 (red), centromere proteins (blue) (**a**,**b**) and MLH1 (green) (**a**,**b**). (**d**–**f**) The same cells after FISH with the pale martin GRC paint probe. Arrowheads point to GRCs. Inserts show zooms at the GRC with enhanced brightness and contrast (**a**–**c**) and schematic representations of GRC SCs (**d**–**f**). Note MLH1 foci at both ends of the partially paired GRC bivalent (**b**). Bar – 5 µm.

GRC univalents in spermatocytes differed in appearance from those in oocytes. Their SCs were longer (24.5 ± 8.5 µm and 19.3 ± 7.0 µm, correspondingly; Mann–Whitney *U* test: p < 0.001) and less intensely labelled with antibodies to SYCP3 (Figs. 4a and 2c). In most spermatocytes, we detected non-specific labeling of anticentromere antibodies over the chromatin of the GRC univalent (Fig. 4a,b). Although the univalents did not display self-synapsis, their proximal and distal ends were usually brought together (Fig. 4a). No MLH1 signals were detected at the univalents.

Two GRCs in the spermatocytes of the mosaic males were usually paired at their proximal ends and rarely at the both ends (Fig. 4b,e). The medial parts of GRC bivalents were always unpaired. Most bivalents did not show MLH1 signals. GRC bivalents in spermatocytes were shorter than univalents (17.4 ± 3.8 µm vs 24.5 ± 8.5 µm; Mann–Whitney *U* test: p < 0.001). We observed MLH1 foci at GRC bivalents in one mosaic male only (Table 2, #2). When a single MLH1 focus was observed, it was always located at the proximal end, while when two MLH1 foci were observed, they were located at both ends of the bivalent (Fig. 4b).

**Figure 5:**
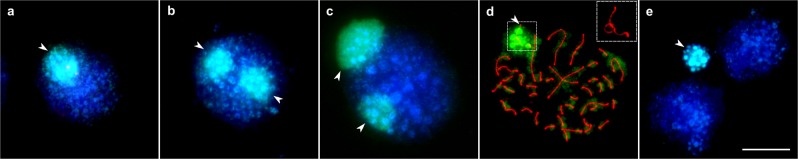
Visualization of GRCs on the spreads of pale martin germ cells after FISH with the pale martin GRC probe (green), and DAPI staining (blue). (**a**–**c**) Spermatogonia with one (**a**) and two (**b**,**c**) GRC copies. (**d**) Pachytene spermatocyte immunolabelled with antibodies against SYCP3 (red) with one GRC copy. Insert shows zoom at the GRC with enhanced brightness and contrast. (**e**) Post-meiotic cells and ejected GRC as round chromatin body. Arrowheads point to GRCs. Bar – 10 µm.

We used a DNA probe specific for the pale martin GRC to analyze GRC number in pale martin germ cells at sequential stages of spermatogenesis. All spermatogonia identified by their size (the nuclear diameter being about 13–15 µm) showed at least one hybridization signal (Fig. 5a–c). About 11% of them contained two GRC signals (Fig. 5b,c) (Table 2). In some cells, one GRC remained in the nuclei, while the other was buckled out or lay close to the nuclei (Fig. 5c).

All pachytene spermatocytes contained one diffuse hybridization signal over the GRC in the case of one or two GRC copies (Figs. 4d,e and 5d) or two signals in the case of three GRC copies (Fig. 4f). More advanced spermatocyte nuclei (with SCs already dissembled) showed no hybridization signal. Near some of these nuclei, we observed round dense chromatin bodies producing a strong homogeneous hybridization signal (Fig. 5e). We did not detect hybridization signals at spermatids and spermatozoa. This indicates that no GRC is present in post-meiotic male germ cells.

None of the four zebra finch males used in this study was mosaic for GRC copy number. All of them had GRC univalents in all four hundred pachytene cells examined (about 100 cells per individual). FISH with the DNA probe specific for the zebra finch GRC showed that all their spermatogonia and all their primary spermatocytes had a single hybridization signal.

## Discussion

The martins and estrildid finches show a striking similarity in morphology and meiotic behavior of their GRCs despite the dozens of million years of divergence and a low homology between them^5^. In both bird lineages, the GRCs appear as large acrocentric macrochromosomes and are usually present in two copies in female and in one copy in male pachytene cells. Even the proportion of females with one GRC copy is the same in the sand martins (Table 1) and zebra finches^2^: about one tenth. In martin and zebra finch females, GRC bivalents synapse along their whole length, but recombine predominately at their ends (Figs. 2a and 3). In both species, sperm cells do not contain GRC. It is transmitted through females only.

Pigozzi and Solari^3^ proposed that all zygotes receive a single maternally derived GRC. They presumed that GRC chromatid nondisjunction occurs during germ line/soma differentiation resulting in germ line cells with two GRCs and in somatic cells with none. To explain why male zebra finches always have one GRC, they suggested lagging of one of the GRC copies during the mitotic divisions of spermatogonia.

This scenario contains at least two unsupported assumptions. First, although the mechanisms of primordial germ cell specification in birds is controversial, it is clear that there is no specific point of “germ line/soma differentiation”. Apparently, the primordial germ cells are developed from pluripotent cells in the maternally specified embryonic region due to induction by factors transiently secreted by adjacent cells^13^. Second, as far as we know, a preferential chromosome segregation in mitotic divisions has never been described.

**Figure 6:**
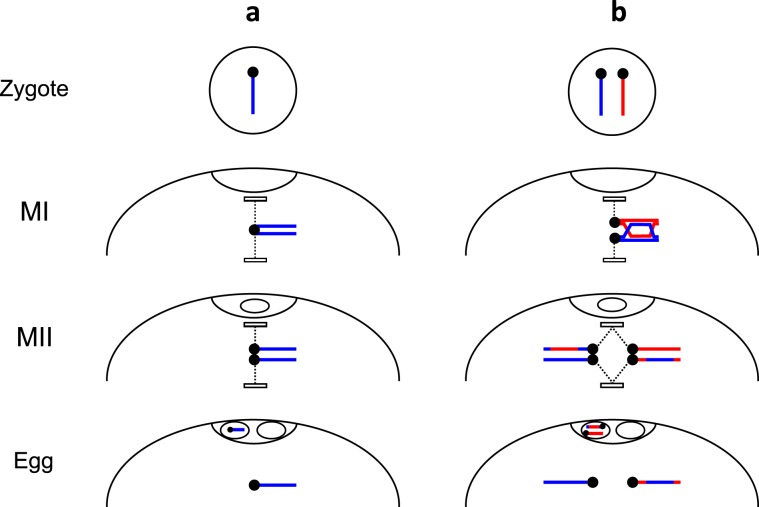
Scenario of GRC transmission. (**a**) Single GRC, containing multiple copies of pericentromeric repeats, remains in the oocyte during MI. Its sister GRCs segregate at MII, producing egg with one GRC (**b**). Double GRC synapses and recombines in their terminal regions. The GRC bivalent remains in the oocyte during MI. During MII, two pairs of the GRC disjoin independently, producing egg with two GRCs.

We propose an alternative scenario of GRC transmission (Fig. 6) based on an assumption of meiotic drive: preferential segregation of two GRCs into the egg during the first meiotic division (MI). Meiotic drive has been described for B-chromosomes, sex chromosomes and various chromosome rearrangements in many species^14–18^.

Under this scenario, the number of GRCs does not change in the female germ line from zygote to egg. If one GRC is present, it remains in the oocyte after MI. During the second division (MII), one of the sister GRCs segregate into the second polar body, other to the egg (Fig. 6a). If two GRCs are present, the both stay in the oocyte in MI. During MII, two pairs of sister GRCs segregate orderly, producing an egg with two GRCs (Fig. 6b). The resulted eggs are fertilized by sperm containing no GRC.

There is no sex difference among the zygotes in the number of GRC: in the martins, the frequency of single GRC females and males is approximately the same (Tables 1 and 2). Somatic cells passively loose or actively eject GRC during early ontogenesis, while primordial germ cells faithfully reproduce zygotic GRC copy number. Male germ line cells with two GRCs successively loose them: one of GRCs might be lost or ejected during spermatogonial proliferation while the other – during the first meiotic division (Fig. 4). Three GRC copies in a pachytene spermatocyte can occur as a rare result of GRC nondisjunction during mitotic divisions of a spermatogonium. We have not observed mosaic females. This indicates that female germ cells stably reproduce zygotic GRC karyotype.

Although this scenario is rather speculative, several features of meiotic behavior, genetic content and morphology common for the GRCs of zebra finches and martins support the assumption that meiotic drive of GRC might take place in the female germ line.

Earlier Torgasheva *et al*.^5^ demonstrated that GRC contains multiple copies of the sequences homologous to pericentromeric repeats. FISH with a pale martin GRC-specific probe produced hybridization signals in the pericentromeric regions of the autosomes, which were diminished after suppression with Cot-1 DNA. The abundance of pericentromeric satellite repetitive DNA in the GRC may contribute to the “centromere drive”^19,20^: an increase of probability of GRC segregation into the oocyte with increased number of microtubules attached.

In martins and estrildid finches, the GRC is one of the largest macrochromosomes. This can facilitate meiotic drive, because the longer the chromosome, the stronger the “polar wind” – the attraction of the kinetochore to the pole in its close vicinity^21^.

The polarized recombination pattern along the GRC bivalents in the martin and zebra finch females (Figs. 2a and 3) can contribute to GRC non-disjunction during MI. It has been shown in yeast, fruit flies and humans that chiasmata located too distally or too proximally on a bivalent are associated with increased frequency of meiotic non-disjunction^22–24^. A non-disjoined bivalent with a double dose of centromeres has a high chance of attaching an increased number of microtubules and remaining in the oocyte. Therefore, a polarized pattern of the MLH1 focus distribution along the GRC bivalent in the females might be considered as an adaptation for its obligatory non-disjunction in MI and transmission of both GRC copies via the maternal line.

Several problems of GRC transmission in meiosis and germline mitosis remain unresolved.

Our scenario suggests the same proportion of the males and females with one GRC in the germ lines. The rest of the males should be mosaics. Yet, no mosaic males have been found in the zebra finch. We may suggest that they lose GRC very early. Study on large sample of zebra finch males is necessary to verify this suggestion.

Another problem is the cause of polymorphism for GRC number in the martins and estrildid finches. Is it neutral or maintained by a balanced selection? Normal GRC segregation in MI of double GRC females would produce 50% of single GRC eggs. Single GRC females, in the case of random GRC segregation, would generate 50% of eggs without GRC, which are apparently unviable, since no bird without GRC has been observed. Such a balance between generation and elimination of single GRC females could be a reason for the stable polymorphism. Alternatively or additionally, the balance between single and double GRC individuals might be maintained due to occasional GRC loss or duplication during mitotic divisions in female germ line.

Unfortunately, cytological analysis of meiotic divisions in female birds is complicated, because these divisions occur 1–2 hours before ovulation, the time of which is impossible to predict. Therefore, our scenario of GRC transmission cannot be tested directly. However, sequencing and assembling of GRC of songbirds and future analysis of genetic variation of GRC linked markers might shed a light on the transmission and evolution of this remarkable chromosome. Sand martins and pale martins with their huge breeding colonies provide a good model for this study.

## Materials and Methods

### Experimental model and subject details

We examined 24 sand martin females, three pale martin females, and nine pale martin males. Adult males were captured at the beginning of the breeding season during the last week of May near bird nests. Nestling females on days 3–6 after hatching were collected from the nests. The number of individuals examined is shown in Tables 1 and 2. The sampling localities are shown in Supplementary Table S1. We also analyzed four adult zebra finch males purchased from pet shops.

The martin species were identified morphologically and by DNA barcoding. DNA was extracted from heart and kidney tissue samples by the conventional phenol-chloroform technique. Primers and PCR conditions for the amplification of a fragment of the mitochondrial COI gene were as described by Hebert *et al*.^25^. The PCR products were separated by electrophoresis in 1% agarose gel, cut from the gel and extracted using a commercial DNA gel extraction kit (BioSilica, Novosibirsk, Russia). The amplicons were Sanger sequenced using the BigDye3.1 reagent (ThermoFisher Scientific, USA), and the sequence was processed using MEGA7 (https://megasoftware.net). The sequence was then analyzed using the distance-based and tree-based identification tools of the BOLD v.4 database http://boldsystems.org^26^. The DNA sequences confirmed correct identification of the individuals as being the sand martin (*R. riparia*) (GenBank accession number MN216344) and the pale martins (*R. diluta*) (GenBank accession number MN216343) according to Pavlova *et al*.^9^

Capture, handling and euthanasia of the birds followed the protocols approved by the Animal Care and Use Committee of the Institute of Cytology and Genetics SD RAS (protocol #35 from 26.10.2016). Experiments described in this manuscript were carried out in accordance with the approved national guidelines for the care and use of animals.

### Mitotic chromosome spreading and staining

Mitotic chromosome spreads were prepared from short-term bone marrow cell cultures incubated in Dulbecco’s Modified Eagle’s medium with 10 µg/ml colchicine for 2 h at 37 °C. The cells were swollen in 0.56% KCI, fixed in methanol-acetic acid (3:1), and spread by air-drying on a microscope slide. Chromosomes were stained with Giemsa.

### SC spreading, immunostaining and FISH with a GRC-specific DNA probe

Chromosome spreads for SC analysis were prepared from testes and ovaries by the drying down method^27^. For electron microscopic examination, the spreads were stained with silver nitrate^28^, covered with a plastic film and transferred to specimen grids. Immunostaining was performed according to the protocol described by Anderson *et al*.^29^ using rabbit polyclonal anti-SYCP3 (1:500; Abcam), mouse monoclonal anti-MLH1 (1:50; Abcam), and human anticentromere (ACA) (1:100; Antibodies Inc) primary antibodies. The secondary antibodies used were Cy3-conjugated goat anti-rabbit (1:500; Jackson ImmunoResearch), FITC-conjugated goat anti-mouse (1:50; Jackson ImmunoResearch), and AMCA-conjugated donkey anti-human (1:100; Jackson ImmunoResearch). Antibodies were diluted in PBT (3% bovine serum albumin and 0.05% Tween 20 in phosphate-buffered saline). A solution of 10% PBT was used for blocking. Primary antibody incubations were performed overnight in a humid chamber at 37 °C; and secondary antibody incubations, for 1 h at 37 °C. Slides were mounted in Vectashield antifade mounting medium (Vector Laboratories, USA) to reduce fluorescence fading.

DNA probes derived from GRCs of the pale martin males and the zebra finch males were prepared as previously described^5^. FISH experiments with these probes on the SC spreads were performed according to a standard protocol with salmon sperm DNA (Ambion, USA) as a DNA carrier. Chromosomes were counterstained with DAPI dissolved in Vectashield antifade solution (Vector Laboratories, USA).

### Microscopic analysis

Images of DAPI-stained metaphase chromosomes and SC spreads after immunostaining and FISH were captured using a CCD-camera installed on an Axioplan 2 compound microscope (Carl Zeiss, Germany) equipped with filter cubes #49, #10, and #15 (ZEISS, Germany) using ISIS4 (METASystems GmbH, Germany). The location of each captured immunostained spread was recorded so that it could be relocated on the slide after FISH. Electron microscopy was carried out using a JEM-1400 electron microscope (JEOL, Tokyo, Japan) at 80 kV. All microscopy studies were carried out at the Center for Microscopic Analysis of Biological Objects of SD RAS (Novosibirsk, Russia). Corel PaintShop Pro X6 (Corel) was used for a correction of image brightness and contrast.

### Chromosome measurements and generation of recombination maps of GRCs

Centromeres were identified by ACA foci. MLH1 signals were only scored if they were localized on SCs. The length of the SC was measured in micrometers and the positions of MLH1 foci in relation to the centromere were recorded using MicroMeasure 3.3^30^. SCs of GRC and macrochromosomes were identified by their relative lengths and centromeric indexes. SC1 is the largest submetacentric. SC2 and SC3 are large subacrocentrics of similar sizes but different centromeric indexes. SC4 is middle size metacentric, SC5 and SC6 are subacrocentrics of the same size, which differ from each other in the centromeric indexes. On bone marrow metaphase chromosome spreads, Z and W are identified as a pair of non-matching macrochromosomes: metacentric and submetacentric (correspondingly). At SC spreads, ZW is identified as macrobivalent with misaligned centromeres and/or asynapsed ends of the axial elements. GRC is identified as the only acrocentric macrobivalent or univalent. To generate recombination maps, we divided the length of the SC into equal intervals approximately equal to 1 µm and plotted the proportion of MLH1 foci located in each interval. STATISTICA 6.0 software package (StatSoft, Tulsa, OK, USA) was used for descriptive statistics. All results were expressed as mean ± SD; p < 0.05 was considered as statistically significant.

## Supplementary information

Supplementary Information.
