# Supplementary material for: Germline-restricted chromosome (GRC) in the sand martin and the pale martin (Hirundinidae, Aves): synapsis, recombination and copy number variation
Source: Sci Rep. 2020 Jan 23;10:1058. doi: 10.1038/s41598-020-58032-4 (PMC6978364; doi:10.1038/s41598-020-58032-4)
Supplement: Supplementary file 1 — Supplementary Information. [file 41598_2020_58032_MOESM1_ESM.pdf]

Germline-restricted chromosome (GRC) in the sand martin and the pale martin (Hirundinidae, Aves): synapsis, recombination and copy number variation

Lyubov P. Malinovskaya[1,2], Kira S. Zadesenets[1], Tatyana V. Karamysheva[1], Ekaterina A. Akberdina[1], Elena A. Kizilova[1,2], Margarita V. Romanenko[2], Elena P. Shnaider[3], Mariya M. Scherbakova[4], Igor G. Korobitsyn[4], Nikolai B. Rubtsov[1,2], Pavel M. Borodin[1,2] and Anna A. Torgasheva[1, 2,*]

[1] Institute of Cytology and Genetics, Russian Academy of Sciences, Siberian Department, 630090 Novosibirsk, Russia; malinovskaya@bionet.nsc.ru (L.P.M); kira_z@bionet.nsc.ru (K.S.Z.) kary@bionet.nsc.ru (T.V.K); rubt@bionet.nsc.ru (N.B.R.); borodin@bionet.nsc.ru (P.M.B.); torgasheva@bionet.nsc.ru (A.A.T.)

[2] Novosibirsk State University, Novosibirsk, 630090, Russia; m.romanenko1@g.nsu.ru (M.V.R)

[3] SibEcoCenter LLC, Novosibirsk, 630009, Russia; lenapshneider17@gmail.com (E.P.S.)

[4] Tomsk State University, Tomsk, 634050, Russia; mary_scherbakova@yahoo.com (M.M.S.); rozenpom@mail.ru (I.G.K.)

***Correspondence to torgasheva@bionet.nsc.ru**

RUNNING TITLE: Germline-restricted chromosome (GRC) in sand and pale martins

ADDITIONAL KEYWORDS: meiosis, germ line, chromatin diminution, programmed DNA elimination, bird genome, chromosome evolution

Supplementary Table S1. Sampling localities

Locality	Latitude	Longitude	Species	N females	N males	Nest ID	Specimen ID
Karasuk	53°43’36" N	77°50’22" E	Sand martin	8	-	3, 11, 12	
Berdsk	54°46'35" N	83°06'06" E	Sand martin	6	-	1, 7, 10	
Baryshevo	54°57'30" N	83°10'21" E	Sand martin	3	-	4, 9	
Tomsk	56°26'49" N	84°56'59" E	Sand martin	7	-	2, 5, 6, 8	
Baryshevo	54°57'30" N	83°10'21" E	Pale martin	-	3		7, 8, 9
Tomsk	56°26'49" N	84°56'59" E	Pale martin	3	6	13,14	1, 2, 3, 4, 5, 6

Supplementary Data. SC length and MLH1 foci number at GRC of sand and pale martins

Species	Gender	№ individual	№ photo	SC length, µm	MLH1 foci number	GRC form
R. riparia	female	riparia1F	4	10.3	2	bivalent
R. riparia	female	riparia1F	13	11.5	2	bivalent
R. riparia	female	riparia1F	15	9.1	2	bivalent
R. riparia	female	riparia1F	3	12.6	1	bivalent
R. riparia	female	riparia1F	6	9.4	1	bivalent
R. riparia	female	riparia1F	7	8.8	1	bivalent
R. riparia	female	riparia1F	9	7.7	2	bivalent
R. riparia	female	riparia1F	10	10.0	2	bivalent
R. riparia	female	riparia1F	12	10.5	2	bivalent
R. riparia	female	riparia1F	18	9.6	2	bivalent
R. riparia	female	riparia1F	20	12.1	1	bivalent
R. riparia	female	riparia1F	22	9.2	1	bivalent
R. riparia	female	riparia1F	25	9.3	1	bivalent
R. riparia	female	riparia1F	26	9.9	2	bivalent
R. riparia	female	riparia1F	27	11.2	2	bivalent
R. riparia	female	riparia1F	28	8.9	2	bivalent
R. riparia	female	riparia1F	30	10.6	2	bivalent
R. riparia	female	riparia1F	31	10.6	2	bivalent
R. riparia	female	riparia2F	6	11.7	2	bivalent
R. riparia	female	riparia2F	8	8.7	2	bivalent
R. riparia	female	riparia2F	10	9.8	2	bivalent
R. riparia	female	riparia2F	11	20.4	2	bivalent
R. riparia	female	riparia2F	12	9.2	2	bivalent
R. riparia	female	riparia2F	13	9.1	2	bivalent
R. riparia	female	riparia2F	15	12.7	2	bivalent
R. riparia	female	riparia2F	16	10.8	2	bivalent
R. riparia	female	riparia2F	18	12.2	2	bivalent
R. riparia	female	riparia2F	20	13.0	2	bivalent
R. riparia	female	riparia2F	22	10.9	2	bivalent
R. riparia	female	riparia2F	23	12.0	2	bivalent
R. riparia	female	riparia2F	24	9.2	2	bivalent
R. riparia	female	riparia2F	25	10.5	1	bivalent
R. riparia	female	riparia2F	26	10.0	2	bivalent
R. riparia	female	riparia2F	27	8.7	2	bivalent
R. riparia	female	riparia2F	28	15.7	2	bivalent
R. riparia	female	riparia2F	29	12.6	2	bivalent
R. riparia	female	riparia2F	30	9.4	2	bivalent
R. riparia	female	riparia2F	31	11.5	2	bivalent
R. riparia	female	riparia2F	32	11.1	2	bivalent
R. riparia	female	riparia2F	33	8.7	2	bivalent
R. riparia	female	riparia2F	34	15.4	2	bivalent
R. riparia	female	riparia2F	35	10.3	2	bivalent
R. riparia	female	riparia2F	36	13.3	2	bivalent
R. riparia	female	riparia2F	37	11.9	2	bivalent
R. riparia	female	riparia2F	38	15.0	2	bivalent

R. riparia	female	riparia2F	39	12.9	1	bivalent
R. riparia	female	riparia2F	40	11.4	2	bivalent
R. riparia	female	riparia2F	41	10.1	2	bivalent
R. riparia	female	riparia2F	50	10.0	2	bivalent
R. riparia	female	riparia2F	51	9.0	2	bivalent
R. riparia	female	riparia2F	52	12.6	2	bivalent
R. riparia	female	riparia2F	55	12.4	2	bivalent
R. riparia	female	riparia2F	58	9.2	2	bivalent
R. riparia	female	riparia2F	61	9.7	2	bivalent
R. riparia	female	riparia2F	63	11.4	2	bivalent
R. riparia	female	riparia2F	64	11.9	1	bivalent
R. riparia	female	riparia2F	67	9.2	2	bivalent
R. riparia	female	riparia2F	68	11.3	2	bivalent
R. riparia	female	riparia2F	69	9.6	2	bivalent
R. riparia	female	riparia2F	70	10.4	2	bivalent
R. riparia	female	riparia2F	71	7.7	2	bivalent
R. riparia	female	riparia2F	72	11.8	2	bivalent
R. riparia	female	riparia2F	73	9.5	2	bivalent
R. riparia	female	riparia2F	75	9.5	2	bivalent
R. riparia	female	riparia2F	77	12.7	2	bivalent
R. riparia	female	riparia2F	79	11.8	2	bivalent
R. riparia	female	riparia2F	80	10.3	2	bivalent
R. riparia	female	riparia2F	81	12.2	2	bivalent
R. riparia	female	riparia2F	82	10.3	2	bivalent
R. riparia	female	riparia2F	83	9.8	2	bivalent
R. riparia	female	riparia2F	84	12.8	2	bivalent
R. riparia	female	riparia2F	85	9.6	2	bivalent
R. riparia	female	riparia2F	87	10.5	2	bivalent
R. riparia	female	riparia2F	88	11.9	2	bivalent
R. riparia	female	riparia2F	90	9.3	2	bivalent
R. riparia	female	riparia2F	92	9.2	2	bivalent
R. riparia	female	riparia2F	93	16.3	2	bivalent
R. riparia	female	riparia2F	94	11.4	2	bivalent
R. riparia	female	riparia2F	95	10.7	2	bivalent
R. riparia	female	riparia2F	96	9.5	2	bivalent
R. riparia	female	riparia2F	97	9.5	1	bivalent
R. riparia	female	riparia2F	98	15.1	2	bivalent
R. riparia	female	riparia2F	99	10.4	2	bivalent
R. riparia	female	riparia2F	100	11.4	2	bivalent
R. riparia	female	riparia2F	101	10.1	2	bivalent
R. riparia	female	riparia2F	102	9.7	2	bivalent
R. riparia	female	riparia2F	103	12.3	2	bivalent
R. riparia	female	riparia2F	104	10.0	2	bivalent
R. riparia	female	riparia2F	105	11.3	2	bivalent
R. riparia	female	riparia2F	110	13.5	2	bivalent
R. riparia	female	riparia2F	112	11.9	2	bivalent
R. riparia	female	riparia2F	113	14.0	2	bivalent
R. riparia	female	riparia2F	114	8.3	2	bivalent

R. riparia	female	riparia2F	115	8.5	2	bivalent
R. riparia	female	riparia2F	117	18.4	2	bivalent
R. riparia	female	riparia2F	118	9.6	2	bivalent
R. riparia	female	riparia2F	119	11.4	2	bivalent
R. riparia	female	riparia2F	120	13.5	2	bivalent
R. riparia	female	riparia2F	121	10.8	2	bivalent
R. riparia	female	riparia2F	122	12.0	2	bivalent
R. riparia	female	riparia2F	123	11.2	2	bivalent
R. riparia	female	riparia2F	124	10.4	2	bivalent
R. riparia	female	riparia2F	125	10.4	2	bivalent
R. riparia	female	riparia2F	126	9.5	2	bivalent
R. riparia	female	riparia2F	127	7.9	1	bivalent
R. riparia	female	riparia2F	128	10.4	2	bivalent
R. riparia	female	riparia2F	129	10.4	2	bivalent
R. riparia	female	riparia2F	130	7.4	2	bivalent
R. riparia	female	riparia2F	132	10.6	2	bivalent
R. riparia	female	riparia2F	133	11.5	2	bivalent
R. riparia	female	riparia2F	134	10.0	2	bivalent
R. riparia	female	riparia2F	135	10.6	2	bivalent
R. riparia	female	riparia2F	136	7.1	2	bivalent
R. riparia	female	riparia2F	137	7.2	2	bivalent
R. riparia	female	riparia2F	138	10.9	2	bivalent
R. riparia	female	riparia2F	139	12.2	2	bivalent
R. riparia	female	riparia2F	140	11.2	2	bivalent
R. riparia	female	riparia2F	141	10.1	2	bivalent
R. riparia	female	riparia2F	142	10.7	1	bivalent
R. riparia	female	riparia2F	143	12.6	2	bivalent
R. riparia	female	riparia2F	144	10.8	2	bivalent
R. riparia	female	riparia2F	145	11.8	2	bivalent
R. riparia	female	riparia2F	146	9.6	2	bivalent
R. riparia	female	riparia3F	3	14.1	2	bivalent
R. riparia	female	riparia3F	4	14.0	2	bivalent
R. riparia	female	riparia3F	6	16.9	2	bivalent
R. riparia	female	riparia3F	7	13.7	2	bivalent
R. riparia	female	riparia3F	8	19.9	2	bivalent
R. riparia	female	riparia3F	11	14.9	2	bivalent
R. riparia	female	riparia3F	13	16.3	2	bivalent
R. riparia	female	riparia3F	16	18.1	2	bivalent
R. riparia	female	riparia3F	17	16.8	3	bivalent
R. riparia	female	riparia3F	18	14.7	2	bivalent
R. riparia	female	riparia3F	19	18.0	2	bivalent
R. riparia	female	riparia3F	20	16.9	2	bivalent
R. riparia	female	riparia3F	1	13.4	2	bivalent
R. riparia	female	riparia3F	2	20.1	2	bivalent
R. riparia	female	riparia3F	5	14.1	2	bivalent
R. riparia	female	riparia3F	6	21.2	2	bivalent
R. riparia	female	riparia3F	9	17.8	2	bivalent
R. riparia	female	riparia3F	11	17.2	2	bivalent

R. riparia	female	riparia3F	13	15.5	2	bivalent
R. riparia	female	riparia3F	14	22.0	2	bivalent
R. riparia	female	riparia3F	15	16.7	2	bivalent
R. riparia	female	riparia3F	16	23.5	2	bivalent
R. riparia	female	riparia3F	18	12.8	2	bivalent
R. riparia	female	riparia3F	19	33.4	2	bivalent
R. riparia	female	riparia3F	20	16.5	2	bivalent
R. riparia	female	riparia3F	21	15.9	2	bivalent
R. riparia	female	riparia3F	22	18.0	2	bivalent
R. riparia	female	riparia3F	23	20.2	2	bivalent
R. riparia	female	riparia3F	24	18.0	2	bivalent
R. riparia	female	riparia3F	25	11.3	2	bivalent
R. riparia	female	riparia3F	27	16.8	2	bivalent
R. riparia	female	riparia3F	28	17.4	2	bivalent
R. riparia	female	riparia3F	29	38.0	2	bivalent
R. riparia	female	riparia3F	32	15.9	2	bivalent
R. riparia	female	riparia3F	33	13.6	2	bivalent
R. riparia	female	riparia3F	34	16.0	2	bivalent
R. riparia	female	riparia3F	38	15.5	2	bivalent
R. riparia	female	riparia3F	40	17.0	2	bivalent
R. riparia	female	riparia3F	41	20.7	2	bivalent
R. riparia	female	riparia4F	1	9.1	2	bivalent
R. riparia	female	riparia4F	4	11.4	2	bivalent
R. riparia	female	riparia4F	5	10.6	2	bivalent
R. riparia	female	riparia4F	11	9.2	2	bivalent
R. riparia	female	riparia4F	14	11.1	2	bivalent
R. riparia	female	riparia4F	22	9.3	2	bivalent
R. riparia	female	riparia4F	23	8.9	2	bivalent
R. riparia	female	riparia4F	25	14.2	2	bivalent
R. riparia	female	riparia4F	29	19.9	2	bivalent
R. riparia	female	riparia4F	5	9.8	2	bivalent
R. riparia	female	riparia4F	9	11.9	2	bivalent
R. riparia	female	riparia4F	10	10.9	2	bivalent
R. riparia	female	riparia4F	12	13.0	2	bivalent
R. riparia	female	riparia4F	14	10.2	2	bivalent
R. riparia	female	riparia4F	22	9.0	2	bivalent
R. riparia	female	riparia4F	24	9.2	2	bivalent
R. riparia	female	riparia4F	25	8.6	2	bivalent
R. riparia	female	riparia4F	27	9.4	2	bivalent
R. riparia	female	riparia4F	31	10.1	2	bivalent
R. riparia	female	riparia4F	33	8.8	2	bivalent
R. riparia	female	riparia4F	36	9.0	2	bivalent
R. riparia	female	riparia4F	38	9.6	2	bivalent
R. riparia	female	riparia4F	39	8.1	2	bivalent
R. riparia	female	riparia4F	40	10.2	2	bivalent
R. riparia	female	riparia4F	42	10.4	2	bivalent
R. riparia	female	riparia4F	46	8.6	2	bivalent
R. riparia	female	riparia4F	51	9.0	2	bivalent

R. riparia	female	riparia4F	52	8.8	2	bivalent
R. riparia	female	riparia4F	53	7.9	2	bivalent
R. riparia	female	riparia4F	56	14.8	2	bivalent
R. riparia	female	riparia4F	57	8.6	2	bivalent
R. riparia	female	riparia4F	58	9.1	2	bivalent
R. riparia	female	riparia4F	68	10.3	2	bivalent
R. riparia	female	riparia5F	5	15.5	3	bivalent
R. riparia	female	riparia5F	10	17.8	2	bivalent
R. riparia	female	riparia5F	11	18.3	2	bivalent
R. riparia	female	riparia5F	13	15.1	2	bivalent
R. riparia	female	riparia5F	14	14.4	2	bivalent
R. riparia	female	riparia5F	17	17.6	2	bivalent
R. riparia	female	riparia5F	20	16.5	2	bivalent
R. riparia	female	riparia5F	23	16.3	2	bivalent
R. riparia	female	riparia5F	27	19.4	2	bivalent
R. riparia	female	riparia5F	29	21.3	3	bivalent
R. riparia	female	riparia5F	3	11.0	2	bivalent
R. riparia	female	riparia6F	3	11.9	2	bivalent
R. riparia	female	riparia6F	4	12.2	2	bivalent
R. riparia	female	riparia6F	6	13.3	2	bivalent
R. riparia	female	riparia6F	8	10.9	2	bivalent
R. riparia	female	riparia6F	9	12.3	2	bivalent
R. riparia	female	riparia6F	10	18.4	2	bivalent
R. riparia	female	riparia6F	11	12.6	2	bivalent
R. riparia	female	riparia6F	12	14.6	2	bivalent
R. riparia	female	riparia6F	13	11.7	2	bivalent
R. riparia	female	riparia6F	14	16.5	2	bivalent
R. riparia	female	riparia6F	15	13.4	2	bivalent
R. riparia	female	riparia6F	16	13.4	2	bivalent
R. riparia	female	riparia6F	17	16.2	2	bivalent
R. riparia	female	riparia6F	18	14.0	2	bivalent
R. riparia	female	riparia6F	19	11.6	2	bivalent
R. riparia	female	riparia6F	20	32.1	2	bivalent
R. riparia	female	riparia6F	21	11.5	2	bivalent
R. riparia	female	riparia6F	22	13.1	2	bivalent
R. riparia	female	riparia7F	1	22.5	1	bivalent
R. riparia	female	riparia7F	2	13.3	2	bivalent
R. riparia	female	riparia7F	4	17.1	1	bivalent
R. riparia	female	riparia7F	5	14.0	1	bivalent
R. riparia	female	riparia7F	8	19.8	2	bivalent
R. riparia	female	riparia7F	9	10.8	2	bivalent
R. riparia	female	riparia7F	14	15.0	2	bivalent
R. riparia	female	riparia7F	15	20.0	2	bivalent
R. riparia	female	riparia7F	16	16.9	2	bivalent
R. riparia	female	riparia7F	17	15.4	2	bivalent
R. riparia	female	riparia7F	19	18.8	1	bivalent
R. riparia	female	riparia7F	20	22.9	2	bivalent
R. riparia	female	riparia7F	21	22.7	2	bivalent

R. riparia	female	riparia7F	5	12.7	2	bivalent
R. riparia	female	riparia7F	6	13.7	2	bivalent
R. riparia	female	riparia7F	8	13.5	2	bivalent
R. riparia	female	riparia7F	11	14.7	2	bivalent
R. riparia	female	riparia7F	12	16.8	2	bivalent
R. riparia	female	riparia7F	13	11.5	2	bivalent
R. riparia	female	riparia7F	16	11.2	2	bivalent
R. riparia	female	riparia7F	17	12.7	3	bivalent
R. riparia	female	riparia7F	18	12.1	2	bivalent
R. riparia	female	riparia7F	23	11.7	1	bivalent
R. riparia	female	riparia8F	1	16.7	0	univalent
R. riparia	female	riparia8F	2	20.0	0	univalent
R. riparia	female	riparia8F	3	17.9	0	univalent
R. riparia	female	riparia8F	4	19.9	0	univalent
R. riparia	female	riparia8F	40	19.4	0	univalent
R. riparia	female	riparia8F	5	13.6	0	univalent
R. riparia	female	riparia8F	6	18.9	0	univalent
R. riparia	female	riparia8F	8	25.3	0	univalent
R. riparia	female	riparia8F	9	32.5	0	univalent
R. riparia	female	riparia8F	11	33.2	0	univalent
R. riparia	female	riparia8F	12	16.8	0	univalent
R. riparia	female	riparia8F	13	61.4	0	univalent
R. riparia	female	riparia8F	14	25.4	0	univalent
R. riparia	female	riparia8F	15	19.2	0	univalent
R. riparia	female	riparia8F	19	13.6	0	univalent
R. riparia	female	riparia8F	20	23.7	0	univalent
R. riparia	female	riparia8F	21	13.3	0	univalent
R. riparia	female	riparia8F	22	15.2	0	univalent
R. riparia	female	riparia8F	23	29.4	0	univalent
R. riparia	female	riparia8F	24	19.8	0	univalent
R. riparia	female	riparia8F	25	16.0	0	univalent
R. riparia	female	riparia8F	26	17.9	0	univalent
R. riparia	female	riparia8F	27	15.5	0	univalent
R. riparia	female	riparia8F	28	18.2	0	univalent
R. riparia	female	riparia8F	29	14.8	0	univalent
R. riparia	female	riparia8F	30	18.0	0	univalent
R. riparia	female	riparia8F	31	13.7	0	univalent
R. riparia	female	riparia8F	32	17.3	0	univalent
R. riparia	female	riparia8F	38	19.1	0	univalent
R. riparia	female	riparia8F	42	18.5	0	univalent
R. riparia	female	riparia8F	45	17.5	0	univalent
R. riparia	female	riparia8F	46	16.0	0	univalent
R. riparia	female	riparia8F	47	13.7	0	univalent
R. riparia	female	riparia8F	48	14.8	0	univalent
R. riparia	female	riparia8F	49	22.2	0	univalent
R. riparia	female	riparia8F	50	13.8	0	univalent
R. riparia	female	riparia8F	52	21.1	0	univalent
R. riparia	female	riparia8F	53	22.5	0	univalent

R. riparia	female	riparia8F	54	18.5	0	univalent
R. riparia	female	riparia8F	56	13.2	0	univalent
R. riparia	female	riparia8F	58	18.4	0	univalent
R. riparia	female	riparia8F	59	17.8	0	univalent
R. riparia	female	riparia8F	61	14.5	0	univalent
R. riparia	female	riparia8F	62	15.8	0	univalent
R. riparia	female	riparia8F	63	36.0	0	univalent
R. riparia	female	riparia8F	64	32.5	0	univalent
R. riparia	female	riparia8F	66	12.3	0	univalent
R. riparia	female	riparia8F	67	16.5	0	univalent
R. riparia	female	riparia8F	68	14.6	0	univalent
R. riparia	female	riparia8F	71	25.4	0	univalent
R. riparia	female	riparia8F	72	10.2	0	univalent
R. riparia	female	riparia8F	74	15.8	0	univalent
R. riparia	female	riparia8F	75	30.0	0	univalent
R. riparia	female	riparia8F	77	19.6	0	univalent
R. riparia	female	riparia8F	78	15.2	0	univalent
R. riparia	female	riparia8F	79	19.3	0	univalent
R. riparia	female	riparia8F	80	21.7	0	univalent
R. riparia	female	riparia8F	81	17.4	0	univalent
R. riparia	female	riparia8F	82	18.2	0	univalent
R. riparia	female	riparia8F	83	15.6	0	univalent
R. riparia	female	riparia9F	4	24.3	0	univalent
R. riparia	female	riparia9F	6	14.5	0	univalent
R. riparia	female	riparia9F	7	20.5	0	univalent
R. riparia	female	riparia9F	8	22.4	0	univalent
R. riparia	female	riparia9F	10	14.6	0	univalent
R. riparia	female	riparia9F	12	22.0	0	univalent
R. riparia	female	riparia9F	16	15.5	0	univalent
R. riparia	female	riparia9F	17	17.7	0	univalent
R. riparia	female	riparia9F	20	16.0	0	univalent
R. riparia	female	riparia9F	20	16.9	0	univalent
R. riparia	female	riparia9F	21	18.8	0	univalent
R. riparia	female	riparia9F	22	15.0	0	univalent
R. riparia	female	riparia9F	26	17.9	0	univalent
R. riparia	female	riparia9F	26	12.8	0	univalent
R. riparia	female	riparia9F	27	18.2	0	univalent
R. riparia	female	riparia9F	28	29.7	0	univalent
R. riparia	female	riparia9F	29	22.1	0	univalent
R. riparia	female	riparia9F	30	14.5	0	univalent
R. riparia	female	riparia9F	34	13.5	0	univalent
R. riparia	female	riparia9F	35	10.4	0	univalent
R. riparia	female	riparia9F	36	21.4	0	univalent
R. riparia	female	riparia9F	38	14.7	0	univalent
R. riparia	female	riparia9F	39	12.4	0	univalent
R. riparia	female	riparia9F	40	14.3	0	univalent
R. riparia	female	riparia9F	41	24.2	0	univalent
R. riparia	female	riparia9F	45	18.2	0	univalent

R. riparia	female	riparia9F	49	17.0	0	univalent
R. riparia	female	riparia9F	54	17.1	0	univalent
R. riparia	female	riparia9F	6	30.1	0	univalent
R. riparia	female	riparia9F	7	15.6	0	univalent
R. riparia	female	riparia9F	9	22.2	0	univalent
R. riparia	female	riparia9F	13	30.9	0	univalent
R. diluta	female	diluta1F	1	15.2	2	bivalent
R. diluta	female	diluta1F	2	16.1	2	bivalent
R. diluta	female	diluta1F	4	12.0	2	bivalent
R. diluta	female	diluta1F	6	13.6	1	bivalent
R. diluta	female	diluta1F	7	11.2	2	bivalent
R. diluta	female	diluta1F	8	12.9	1	bivalent
R. diluta	female	diluta1F	9	15.6		bivalent
R. diluta	female	diluta1F	10	11.3	2	bivalent
R. diluta	female	diluta1F	11	11.3	2	bivalent
R. diluta	female	diluta1F	12	11.5	2	bivalent
R. diluta	female	diluta1F	13	14.3	2	bivalent
R. diluta	female	diluta1F	14	10.5	2	bivalent
R. diluta	female	diluta1F	15	12.3	2	bivalent
R. diluta	female	diluta1F	16	10.4	2	bivalent
R. diluta	female	diluta1F	18	11.4	2	bivalent
R. diluta	female	diluta1F	19	10.5	2	bivalent
R. diluta	female	diluta1F	20	9.8	2	bivalent
R. diluta	female	diluta1F	23	11.7	2	bivalent
R. diluta	female	diluta1F	24	13.1		bivalent
R. diluta	female	diluta1F	25	9.5	2	bivalent
R. diluta	female	diluta1F	26	10.9	2	bivalent
R. diluta	female	diluta1F	27	10.4	2	bivalent
R. diluta	female	diluta1F	30	10.0	2	bivalent
R. diluta	female	diluta1F	31	11.3	2	bivalent
R. diluta	female	diluta1F	32	12.2	2	bivalent
R. diluta	female	diluta1F	33	10.7	2	bivalent
R. diluta	female	diluta1F	34	13.0	2	bivalent
R. diluta	female	diluta1F	35	11.6	2	bivalent
R. diluta	female	diluta1F	36	12.6	2	bivalent
R. diluta	female	diluta1F	38	13.3	2	bivalent
R. diluta	female	diluta1F	39	11.7	2	bivalent
R. diluta	female	diluta1F	40	12.2	1	bivalent
R. diluta	female	diluta1F	41	12.8	2	bivalent
R. diluta	female	diluta1F	42	11.0	2	bivalent
R. diluta	female	diluta1F	43	13.1	2	bivalent
R. diluta	female	diluta1F	44	11.0	2	bivalent
R. diluta	female	diluta1F	45	13.1	1	bivalent
R. diluta	female	diluta1F	47	11.1	2	bivalent
R. diluta	female	diluta1F	51	12.4	2	bivalent
R. diluta	female	diluta1F	52	11.7	2	bivalent
R. diluta	female	diluta1F	53	11.1	2	bivalent
R. diluta	female	diluta1F	54	10.5	1	bivalent

R. diluta	female	diluta1F	55	10.1		bivalent
R. diluta	female	diluta1F	56	13.8	2	bivalent
R. diluta	female	diluta1F	57	13.8	2	bivalent
R. diluta	female	diluta1F	60	10.9	2	bivalent
R. diluta	female	diluta1F	61	12.5	1	bivalent
R. diluta	female	diluta1F	62	11.4	2	bivalent
R. diluta	female	diluta2F	1	12.0	2	bivalent
R. diluta	female	diluta2F	2	12.6	3	bivalent
R. diluta	female	diluta2F	3	9.7	2	bivalent
R. diluta	female	diluta2F	5	13.7	3	bivalent
R. diluta	female	diluta2F	6	10.7	2	bivalent
R. diluta	female	diluta2F	7	10.5	2	bivalent
R. diluta	female	diluta2F	9	10.1	2	bivalent
R. diluta	female	diluta2F	10	13.0	2	bivalent
R. diluta	female	diluta2F	11	8.9	2	bivalent
R. diluta	female	diluta2F	12	10.4	2	bivalent
R. diluta	female	diluta2F	14	9.8	2	bivalent
R. diluta	female	diluta2F	15	11.4	2	bivalent
R. diluta	female	diluta2F	16	12.1	2	bivalent
R. diluta	female	diluta2F	17	10.2	2	bivalent
R. diluta	female	diluta2F	18	10.9	2	bivalent
R. diluta	female	diluta2F	19	10.2	2	bivalent
R. diluta	female	diluta2F	20	8.0	2	bivalent
R. diluta	female	diluta2F	21	10.7	2	bivalent
R. diluta	female	diluta2F	22	12.2	1	bivalent
R. diluta	female	diluta2F	24	11.1	2	bivalent
R. diluta	female	diluta2F	25	14.5	2	bivalent
R. diluta	female	diluta2F	26	12.9	2	bivalent
R. diluta	female	diluta2F	27	13.4	2	bivalent
R. diluta	female	diluta2F	28	10.8	2	bivalent
R. diluta	female	diluta2F	29	12.8	2	bivalent
R. diluta	female	diluta2F	30	12.6	2	bivalent
R. diluta	female	diluta2F	31	13.8	2	bivalent
R. diluta	female	diluta2F	32	10.3	2	bivalent
R. diluta	female	diluta2F	33	9.8	2	bivalent
R. diluta	female	diluta2F	34	15.7	2	bivalent
R. diluta	female	diluta2F	35	10.2	2	bivalent
R. diluta	female	diluta2F	36	12.6	3	bivalent
R. diluta	female	diluta2F	37	14.5	2	bivalent
R. diluta	female	diluta2F	38	11.8	2	bivalent
R. diluta	female	diluta2F	39	12.2	2	bivalent
R. diluta	female	diluta2F	40	10.0	2	bivalent
R. diluta	female	diluta2F	41	12.2	2	bivalent
R. diluta	female	diluta2F	43	11.2	2	bivalent
R. diluta	female	diluta2F	44	32.5	2	bivalent
R. diluta	female	diluta2F	45	12.1	2	bivalent
R. diluta	female	diluta2F	46	10.7	2	bivalent
R. diluta	female	diluta2F	47	12.1	2	bivalent

R. diluta	female	diluta2F	48	14.2	3	bivalent
R. diluta	female	diluta2F	49	14.9	2	bivalent
R. diluta	female	diluta2F	50	12.8	3	bivalent
R. diluta	female	diluta2F	51	12.6	2	bivalent
R. diluta	female	diluta2F	52	13.6	2	bivalent
R. diluta	female	diluta2F	54	13.8	2	bivalent
R. diluta	female	diluta2F	55	11.4	2	bivalent
R. diluta	female	diluta2F	56	13.7	2	bivalent
R. diluta	female	diluta3F	1	8.9	2	bivalent
R. diluta	female	diluta3F	2	9.4	2	bivalent
R. diluta	female	diluta3F	3	9.5	2	bivalent
R. diluta	female	diluta3F	6	12.7	2	bivalent
R. diluta	female	diluta3F	9	7.6	2	bivalent
R. diluta	female	diluta3F	11	16.9	2	bivalent
R. diluta	female	diluta3F	14	7.6	1	bivalent
R. diluta	female	diluta3F	16	7.5	1	bivalent
R. diluta	female	diluta3F	17	15.6	2	bivalent
R. diluta	female	diluta3F	18	7.7	2	bivalent
R. diluta	female	diluta3F	19	9.4	2	bivalent
R. diluta	female	diluta3F	20	13.5	2	bivalent
R. diluta	female	diluta3F	22	9.2	2	bivalent
R. diluta	female	diluta3F	23	7.5	2	bivalent
R. diluta	female	diluta3F	24	9.7	2	bivalent
R. diluta	female	diluta3F	28	8.7	2	bivalent
R. diluta	female	diluta3F	29	12.1	2	bivalent
R. diluta	female	diluta3F	30	8.1	2	bivalent
R. diluta	female	diluta3F	31	10.9	2	bivalent
R. diluta	female	diluta3F	33	9.4	2	bivalent
R. diluta	female	diluta3F	35	20.0	2	bivalent
R. diluta	female	diluta3F	38	9.0	2	bivalent
R. diluta	female	diluta3F	42	13.9	1	bivalent
R. diluta	female	diluta3F	44	10.2	2	bivalent
R. diluta	female	diluta3F	45	16.2	2	bivalent
R. diluta	female	diluta3F	46	8.2	3	bivalent
R. diluta	female	diluta3F	48	10.6	2	bivalent
R. diluta	female	diluta3F	51	12.3		bivalent
R. diluta	female	diluta3F	53	11.7	2	bivalent
R. diluta	female	diluta3F	55	11.6	1	bivalent
R. diluta	female	diluta3F	57	9.4	2	bivalent
R. diluta	female	diluta3F	59	10.9	2	bivalent
R. diluta	female	diluta3F	63	7.6	1	bivalent
R. diluta	female	diluta3F	64	8.5	2	bivalent
R. diluta	female	diluta3F	66	9.5	2	bivalent
R. diluta	female	diluta3F	67	12.9	2	bivalent
R. diluta	female	diluta3F	68	9.7	2	bivalent
R. diluta	female	diluta3F	70	14.3	2	bivalent
R. diluta	female	diluta3F	71	11.7	1	bivalent
R. diluta	female	diluta3F	72	11.0	1	bivalent

R. diluta	female	diluta3F	73	12.6	2	bivalent
R. diluta	female	diluta3F	74	8.6	1	bivalent
R. diluta	female	diluta3F	75	10.6	2	bivalent
R. diluta	female	diluta3F	76	8.8	3	bivalent
R. diluta	female	diluta3F	79	8.9	2	bivalent
R. diluta	female	diluta3F	80	8.5	2	bivalent
R. diluta	female	diluta3F	81	11.0	2	bivalent
R. diluta	female	diluta3F	82	11.1	1	bivalent
R. diluta	female	diluta3F	83	11.5	2	bivalent
R. diluta	female	diluta3F	84	9.0	2	bivalent
R. diluta	male	diluta2M	1	15.9		univalent
R. diluta	male	diluta2M	2	21.4		univalent
R. diluta	male	diluta2M	3	18.4	2	bivalent
R. diluta	male	diluta2M	4	15.3	1	bivalent
R. diluta	male	diluta2M	5	22.7		univalent
R. diluta	male	diluta2M	6	16.8		univalent
R. diluta	male	diluta2M	7	29.4		univalent
R. diluta	male	diluta2M	8	18.1	1	bivalent
R. diluta	male	diluta2M	9	18.2	1	bivalent
R. diluta	male	diluta2M	10	17.2	1	bivalent
R. diluta	male	diluta2M	14	16.3		univalent
R. diluta	male	diluta2M	14	21.6		univalent
R. diluta	male	diluta2M	14	19.4		univalent
R. diluta	male	diluta2M	15	17.0	1	bivalent
R. diluta	male	diluta2M	16	16.1	1	bivalent
R. diluta	male	diluta2M	17	15.0	2	bivalent
R. diluta	male	diluta2M	18	19.9		univalent
R. diluta	male	diluta2M	19	15.6		univalent
R. diluta	male	diluta2M	20	15.5		univalent
R. diluta	male	diluta2M	21	21.4		univalent
R. diluta	male	diluta2M	22	18.5		univalent
R. diluta	male	diluta2M	23	9.5	1	bivalent
R. diluta	male	diluta2M	23	15.7		univalent
R. diluta	male	diluta2M	23	9.5	1	bivalent
R. diluta	male	diluta2M	24	16.3	1	bivalent
R. diluta	male	diluta2M	25	16.5	1	bivalent
R. diluta	male	diluta2M	26	15.7		univalent
R. diluta	male	diluta2M	28	17.7	1	bivalent
R. diluta	male	diluta2M	29	16.7	1	bivalent
R. diluta	male	diluta2M	30	19.5	1	bivalent
R. diluta	male	diluta2M	31	18.1		univalent
R. diluta	male	diluta2M	33	11.7		univalent
R. diluta	male	diluta2M	34	16.0		univalent
R. diluta	male	diluta2M	35	16.0		univalent
R. diluta	male	diluta2M	36	16.9		univalent
R. diluta	male	diluta2M	37	17.1		univalent
R. diluta	male	diluta2M	38	14.1		bivalent
R. diluta	male	diluta2M	39	16.9		univalent

R. diluta	male	diluta2M	40	15.1	1	bivalent
R. diluta	male	diluta2M	41	15.3		univalent
R. diluta	male	diluta2M	42	17.4		univalent
R. diluta	male	diluta2M	43	17.1		univalent
R. diluta	male	diluta2M	44	13.4		univalent
R. diluta	male	diluta2M	45	22.5	1	bivalent
R. diluta	male	diluta2M	46	17.1		univalent
R. diluta	male	diluta2M	47	20.1		univalent
R. diluta	male	diluta2M	48	13.9		univalent
R. diluta	male	diluta2M	49	17.4		univalent
R. diluta	male	diluta2M	50	15.7	1	bivalent
R. diluta	male	diluta2M	51	24.3	1	bivalent
R. diluta	male	diluta2M	52	16.7		bivalent
R. diluta	male	diluta2M	53	13.5		univalent
R. diluta	male	diluta2M	54	15.9		univalent
R. diluta	male	diluta2M	55	10.7		univalent
R. diluta	male	diluta2M	57	16.2	1	bivalent
R. diluta	male	diluta2M	58	23.7		bivalent
R. diluta	male	diluta2M	60	19.6	1	bivalent
R. diluta	male	diluta3M	1	13.0		univalent
R. diluta	male	diluta3M	2	20.8		univalent
R. diluta	male	diluta3M	4	21.9		univalent
R. diluta	male	diluta3M	5	25.2		univalent
R. diluta	male	diluta3M	6	26.6		univalent
R. diluta	male	diluta3M	6	28.9		univalent
R. diluta	male	diluta3M	8	23.2		univalent
R. diluta	male	diluta3M	9	15.8		univalent
R. diluta	male	diluta3M	10	19.6		univalent
R. diluta	male	diluta3M	11	24.5		univalent
R. diluta	male	diluta3M	12	23.9		univalent
R. diluta	male	diluta3M	12	23.5		univalent
R. diluta	male	diluta3M	12	21.9		univalent
R. diluta	male	diluta3M	13	16.4		univalent
R. diluta	male	diluta3M	14	25.0		univalent
R. diluta	male	diluta3M	15	31.4		univalent
R. diluta	male	diluta3M	16	28.1		univalent
R. diluta	male	diluta3M	17	28.9		univalent
R. diluta	male	diluta3M	17	26.9		univalent
R. diluta	male	diluta3M	19	27.9		univalent
R. diluta	male	diluta3M	20	27.7		univalent
R. diluta	male	diluta3M	21	30.6		univalent
R. diluta	male	diluta3M	22	27.2		univalent
R. diluta	male	diluta3M	23	16.1		univalent
R. diluta	male	diluta3M	25	17.3		univalent
R. diluta	male	diluta3M	26	19.2		univalent
R. diluta	male	diluta3M	28	23.1		univalent
R. diluta	male	diluta3M	29	16.8		univalent
R. diluta	male	diluta3M	30	23.0		univalent

R. diluta	male	diluta3M	31	14.9	univalent
R. diluta	male	diluta3M	32	15.7	univalent
R. diluta	male	diluta3M	33	31.9	univalent
R. diluta	male	diluta3M	34	28.7	univalent
R. diluta	male	diluta3M	36	26.1	univalent
R. diluta	male	diluta3M	37	23.8	univalent
R. diluta	male	diluta3M	38	21.7	univalent
R. diluta	male	diluta3M	39	14.1	univalent
R. diluta	male	diluta3M	40	27.5	univalent
R. diluta	male	diluta3M	41	27.8	univalent
R. diluta	male	diluta3M	42	19.2	univalent
R. diluta	male	diluta3M	43	16.6	univalent
R. diluta	male	diluta3M	43	28.2	univalent
R. diluta	male	diluta3M	44	19.7	univalent
R. diluta	male	diluta3M	45	25.2	univalent
R. diluta	male	diluta3M	46	26.1	univalent
R. diluta	male	diluta3M	47	15.7	univalent
R. diluta	male	diluta3M	48	21.0	univalent
R. diluta	male	diluta3M	49	17.5	univalent
R. diluta	male	diluta3M	50	26.6	univalent
R. diluta	male	diluta3M	51	31.7	univalent
R. diluta	male	diluta3M	52	14.7	univalent
R. diluta	male	diluta3M	53	17.1	univalent
R. diluta	male	diluta3M	54	13.9	univalent
R. diluta	male	diluta3M	55	31.5	univalent
R. diluta	male	diluta3M	56	19.4	univalent
R. diluta	male	diluta3M	57	28.8	univalent
R. diluta	male	diluta3M	58	21.6	univalent
R. diluta	male	diluta3M	59	18.6	univalent
R. diluta	male	diluta3M	60	15.1	univalent
R. diluta	male	diluta4M	1	35.6	univalent
R. diluta	male	diluta4M	3	42.0	univalent
R. diluta	male	diluta4M	4	27.7	univalent
R. diluta	male	diluta4M	4	28.2	univalent
R. diluta	male	diluta4M	8	28.4	univalent
R. diluta	male	diluta4M	11	31.3	univalent
R. diluta	male	diluta4M	12	27.5	univalent
R. diluta	male	diluta4M	14	29.4	univalent
R. diluta	male	diluta4M	15	29.8	univalent
R. diluta	male	diluta4M	16	34.3	univalent
R. diluta	male	diluta4M	17	45.7	univalent
R. diluta	male	diluta4M	18	27.0	univalent
R. diluta	male	diluta4M	19	31.7	univalent
R. diluta	male	diluta4M	20	40.7	univalent
R. diluta	male	diluta4M	21	32.9	univalent
R. diluta	male	diluta4M	22	27.2	univalent
R. diluta	male	diluta4M	23	29.1	univalent
R. diluta	male	diluta4M	24	39.7	univalent

R. diluta	male	diluta4M	25	28.0	univalent
R. diluta	male	diluta4M	26	30.0	univalent
R. diluta	male	diluta4M	27	30.0	univalent
R. diluta	male	diluta4M	28	41.9	univalent
R. diluta	male	diluta4M	29	45.4	univalent
R. diluta	male	diluta4M	30	26.2	univalent
R. diluta	male	diluta4M	31	28.3	univalent
R. diluta	male	diluta4M	32	37.4	univalent
R. diluta	male	diluta4M	33	35.3	univalent
R. diluta	male	diluta4M	34	32.0	univalent
R. diluta	male	diluta4M	35	28.7	univalent
R. diluta	male	diluta4M	36	27.6	univalent
R. diluta	male	diluta4M	37	37.3	univalent
R. diluta	male	diluta4M	38	27.7	univalent
R. diluta	male	diluta4M	39	28.7	univalent
R. diluta	male	diluta4M	40	27.5	univalent
R. diluta	male	diluta4M	41	32.4	univalent
R. diluta	male	diluta4M	42	30.0	univalent
R. diluta	male	diluta4M	43	32.9	univalent
R. diluta	male	diluta4M	44	37.4	univalent
R. diluta	male	diluta4M	45	29.2	univalent
R. diluta	male	diluta4M	46	26.6	univalent
R. diluta	male	diluta4M	47	28.4	univalent
R. diluta	male	diluta4M	49	29.6	univalent
R. diluta	male	diluta4M	50	32.4	univalent
R. diluta	male	diluta4M	51	43.3	univalent
R. diluta	male	diluta4M	52	31.0	univalent
R. diluta	male	diluta4M	53	30.3	univalent
R. diluta	male	diluta4M	54	29.4	univalent
R. diluta	male	diluta4M	55	30.5	univalent
R. diluta	male	diluta4M	56	26.4	univalent
R. diluta	male	diluta4M	57	25.8	univalent
R. diluta	male	diluta4M	58	32.9	univalent
R. diluta	male	diluta4M	59	28.9	univalent
R. diluta	male	diluta4M	60	36.6	univalent
R. diluta	male	diluta7M	1	24.3	univalent
R. diluta	male	diluta7M	2	21.3	univalent
R. diluta	male	diluta7M	3	17.9	univalent
R. diluta	male	diluta7M	3	19.1	univalent
R. diluta	male	diluta7M	4	22.6	univalent
R. diluta	male	diluta7M	5	19.8	univalent
R. diluta	male	diluta7M	7	17.0	univalent
R. diluta	male	diluta7M	10	15.1	univalent
R. diluta	male	diluta7M	10	18.3	univalent
R. diluta	male	diluta7M	11	25.9	univalent
R. diluta	male	diluta7M	11	25.4	univalent
R. diluta	male	diluta7M	12	30.0	univalent
R. diluta	male	diluta7M	13	22.2	univalent

R. diluta	male	diluta7M	14	28.5	univalent
R. diluta	male	diluta7M	15	24.6	univalent
R. diluta	male	diluta7M	16	21.4	univalent
R. diluta	male	diluta7M	17	26.1	univalent
R. diluta	male	diluta7M	17	23.9	univalent
R. diluta	male	diluta7M	18	22.4	univalent
R. diluta	male	diluta7M	19	22.4	univalent
R. diluta	male	diluta7M	20	31.9	univalent
R. diluta	male	diluta7M	21	23.2	univalent
R. diluta	male	diluta7M	21	20.5	univalent
R. diluta	male	diluta7M	22	19.2	univalent
R. diluta	male	diluta7M	22	19.6	univalent
R. diluta	male	diluta7M	23	18.1	univalent
R. diluta	male	diluta7M	23	20.7	univalent
R. diluta	male	diluta7M	24	28.8	univalent
R. diluta	male	diluta7M	25	25.2	univalent
R. diluta	male	diluta7M	26	19.8	univalent
R. diluta	male	diluta7M	27	25.5	univalent
R. diluta	male	diluta7M	28	27.2	univalent
R. diluta	male	diluta7M	29	28.3	univalent
R. diluta	male	diluta7M	30	19.4	univalent
R. diluta	male	diluta7M	31	18.4	univalent
R. diluta	male	diluta7M	32	30.4	univalent
R. diluta	male	diluta7M	33	32.4	univalent
R. diluta	male	diluta7M	33	25.9	univalent
R. diluta	male	diluta7M	34	22.7	univalent
R. diluta	male	diluta7M	35	21.6	univalent
R. diluta	male	diluta7M	36	27.1	univalent
R. diluta	male	diluta7M	37	44.8	univalent
R. diluta	male	diluta7M	38	30.9	univalent
R. diluta	male	diluta7M	39	20.3	univalent
R. diluta	male	diluta7M	40	20.3	univalent
R. diluta	male	diluta7M	41	20.2	univalent
R. diluta	male	diluta7M	42	22.1	univalent
R. diluta	male	diluta7M	43	28.1	univalent
R. diluta	male	diluta7M	44	23.8	univalent
R. diluta	male	diluta7M	45	19.5	univalent
R. diluta	male	diluta7M	46	21.1	univalent
R. diluta	male	diluta7M	47	20.3	univalent
R. diluta	male	diluta7M	48	21.7	univalent
R. diluta	male	diluta7M	49	18.6	univalent
R. diluta	male	diluta7M	50	19.9	univalent
R. diluta	male	diluta7M	51	29.1	univalent
R. diluta	male	diluta8M	1	32.0	univalent
R. diluta	male	diluta8M	1	22.3	univalent
R. diluta	male	diluta8M	3	32.1	univalent
R. diluta	male	diluta8M	4	50.8	univalent
R. diluta	male	diluta8M	5	34.6	univalent

R. diluta	male	diluta8M	6	56.4	univalent
R. diluta	male	diluta8M	7	36.3	univalent
R. diluta	male	diluta8M	8	42.3	univalent
R. diluta	male	diluta8M	9	26.1	univalent
R. diluta	male	diluta8M	10	31.4	univalent
R. diluta	male	diluta8M	11	42.3	univalent
R. diluta	male	diluta8M	12	41.5	univalent
R. diluta	male	diluta8M	12	37.9	univalent
R. diluta	male	diluta8M	13	49.5	univalent
R. diluta	male	diluta8M	14	35.0	univalent
R. diluta	male	diluta8M	15	36.1	univalent
R. diluta	male	diluta8M	17	28.1	univalent
R. diluta	male	diluta8M	18	30.1	univalent
R. diluta	male	diluta8M	19	37.7	univalent
R. diluta	male	diluta8M	20	32.7	univalent
R. diluta	male	diluta8M	20	29.2	univalent
R. diluta	male	diluta8M	21	41.8	univalent
R. diluta	male	diluta8M	22	42.9	univalent
R. diluta	male	diluta8M	23	36.0	univalent
R. diluta	male	diluta8M	24	30.8	univalent
R. diluta	male	diluta9M	1	13.3	univalent
R. diluta	male	diluta9M	4	14.8	univalent
R. diluta	male	diluta9M	5	17.3	univalent
R. diluta	male	diluta9M	7	16.8	univalent
R. diluta	male	diluta9M	8	34.5	univalent
R. diluta	male	diluta9M	10	15.7	univalent
R. diluta	male	diluta9M	11	16.8	univalent
R. diluta	male	diluta9M	12	19.8	univalent
R. diluta	male	diluta9M	13	40.4	univalent
R. diluta	male	diluta9M	14	19.8	univalent
R. diluta	male	diluta9M	15	13.9	univalent
R. diluta	male	diluta9M	16	16.4	univalent
R. diluta	male	diluta9M	17	13.8	univalent
R. diluta	male	diluta9M	18	15.3	univalent
R. diluta	male	diluta9M	19	14.9	univalent
R. diluta	male	diluta9M	20	16.5	univalent
R. diluta	male	diluta9M	21	38.8	univalent
R. diluta	male	diluta9M	22	17.2	univalent
R. diluta	male	diluta9M	23	11.7	univalent
R. diluta	male	diluta9M	24	12.1	univalent
R. diluta	male	diluta9M	25	14.6	univalent
R. diluta	male	diluta9M	25	16.7	univalent
R. diluta	male	diluta9M	27	12.4	univalent
R. diluta	male	diluta9M	29	17.6	univalent
R. diluta	male	diluta9M	30	13.0	univalent
R. diluta	male	diluta9M	32	15.6	univalent
R. diluta	male	diluta9M	33	18.4	univalent
R. diluta	male	diluta9M	34	16.7	univalent

R. diluta	male	diluta9M	35	14.2	univalent
R. diluta	male	diluta9M	36	17.9	univalent
R. diluta	male	diluta9M	38	14.8	univalent
R. diluta	male	diluta9M	39	13.9	univalent
R. diluta	male	diluta9M	40	36.2	univalent
R. diluta	male	diluta9M	41	15.8	univalent
R. diluta	male	diluta9M	41	15.2	univalent
R. diluta	male	diluta9M	42	14.0	univalent
R. diluta	male	diluta9M	43	14.3	univalent
R. diluta	male	diluta9M	45	13.6	univalent
R. diluta	male	diluta9M	47	13.6	univalent
R. diluta	male	diluta9M	48	11.8	univalent
R. diluta	male	diluta9M	49	13.8	univalent
R. diluta	male	diluta9M	50	25.3	bivalent
R. diluta	male	diluta9M	51	14.8	univalent
